# The Landscape of Alternative Splicing Regulating Potassium Use Efficiency in *Nicotiana tabacum*

**DOI:** 10.3389/fpls.2021.774829

**Published:** 2021-11-08

**Authors:** Bing He, Lin Meng, Lina Tang, Weicong Qi, Fengqin Hu, Yuanda Lv, Wenjing Song

**Affiliations:** ^1^Key Laboratory of Tobacco Biology and Processing, Ministry of Agriculture, Tobacco Research Institute, Chinese Academy of Agricultural Sciences, Qingdao, China; ^2^Institute of Germplasm Resources and Biotechnology, Jiangsu Academy of Agricultural Sciences, Nanjing, China; ^3^Tobacco Science Research Institute, Fujian Tobacco Monopoly Administration, Fuzhou, China; ^4^Excellence and Innovation Center, Jiangsu Academy of Agricultural Sciences, Nanjing, China

**Keywords:** potassium, alternative splicing (AS), transcriptional regulation, *Nicotiana tabacum* L., transcription factor

## Abstract

Alternative splicing (AS) occurs extensively in eukaryotes as an essential mechanism for regulating transcriptome complexity and diversity, but the AS landscape regulating potassium (K) use efficiency in plants is unclear. In this study, we performed high-throughput transcriptome sequencing of roots and shoots from allopolyploid *Nicotiana tabacum* under K^+^ deficiency. Preliminary physiological analysis showed that root system architecture was dramatically changed due to potassium deficiency and that IAA content was significantly reduced in root and shoot. AS analysis showed that a total of 28,179 genes exhibited 54,457 AS events, and 1,510 and 1,732 differentially alternatively spliced (DAS) events were identified in shoots and roots under low K^+^ stress. Nevertheless, only 120 DAS events occurred in both shoots and roots, implying that most DAS events were tissue-specific. Both in shoot and the root, the proportion of DAS genes in differentially expressed (DE) genes equaled that in non-DE genes, which indicated that AS might play a unique regulatory role in response to low potassium. Gene ontology analysis further indicated that transcription regulation and AS modulation worked independently in response to low K^+^ stress in tobacco, as their target biological processes were different. Totally 45 DAS transcription factors (TFs) were found, which were involved in 18 TF families. Five Auxin response factor (ARF) TFs were significantly DAS in root, suggesting that response to auxin was probably subject to AS regulation in the tobacco root. Our study shows that AS variation occurs extensively and has a particular regulatory mechanism under K^+^ deficiency in tobacco. The study also links changes in root system architecture with the changes in AS of ARF TFs, which implied the functional significance of these AS events for root growth and architecture.

## Introduction

Potassium (K) is one of the indispensable macronutrients for plant growth and development, which exists in the form of ions in plants. Although K^+^ is not assimilated into organic matter, K^+^ plays a significant role in various critical physiological and biochemical processes (Ragel et al., 2019). K^+^ is related to enzyme activity, turgor pressure maintenance, osmotic adjustment, pH homeostasis, biotic and abiotic stresses response, et al. (Egilla et al., 2005; Sigel and Pyle, 2007; Wang et al., 2013; Ragel et al., 2019). Plants absorb a large amount of K^+^ from the soil to maintain growth and development. However, due to the large-scale agricultural production and leaching loss, a large area of the world’s agricultural land lacks K^+^ (Zörb et al., 2014). K^+^ deficiency has already seriously affected agricultural production (Pettigrew, 2008; Hu et al., 2019). The application of K fertilizer is a common technique for improving crop yield and quality. However, the high input of K fertilizer and the low K use efficiency have increased production costs and caused environmental problems. Thus, Enhancing potassium use efficiency (KUE) in crop plants and developing potassium-efficient crop germplasms is necessary to increase farm income while protecting the environment (Yang et al., 2003; Cao et al., 2007; Damon et al., 2007; Zhao et al., 2014; Song et al., 2018). To investigate the molecular mechanism of low K^+^ stress response will help identify key regulatory factors and pathways and lay the foundation for the crop breeding of K^+^ deficiency tolerance.

Nowadays, multiple K^+^ transport proteins have been identified, performing different functions and playing distinct roles in K^+^ uptake and transport (Véry et al., 2014). AKT1 K^+^ channels and HAK5 K^+^ transporter are the main constituents of the K^+^ uptake system in plant roots (Gierth et al., 2005; Xu et al., 2006). AKT1 is involved in K^+^ uptake at a wide range of external K^+^ concentrations (about 0.01–10 mM) (Hirsch et al., 1998). Previous studies have shown that the regulation of AKT1 is not likely to occur at the transcriptional level (Maathuis et al., 2003; Pilot et al., 2003). Instead, the activity of AKT1 is mainly regulated at the post-translational level, including the interaction with KC1 (a negative regulator of K^+^ inward channels) and CBL-CIPK-mediated phosphorylation and dephosphorylation (Xu et al., 2006; Jeanguenin et al., 2011; Li et al., 2014). The transcription of *HAK5* is significantly induced by K^+^ deficiency in roots, and therefore it is considered a marker gene for low K^+^ response (Gierth et al., 2005; Wang et al., 2021). Several transcription factors that bind to the promoter of *HAK5* have been identified, including RAP2.11, ARF2, DDF2, JLO, TF_IIA, and bHLH121 (Santa-María et al., 2018). RAP2.11 (Related to AP2 11) positively regulates *HAK5* under low K^+^ stress (Kim et al., 2012). ARF2 (auxin response factor 2) is the negative regulator of HAK5, which binds to the AtHAK5 promoter and represses its transcription under K^+^-sufficient conditions. Under low K^+^ conditions, ARF2 abolishes its DNA binding activity, allowing other transcription factors to bind to the HAK5 promoter and induce its transcription (Zhao et al., 2016; Santa-María et al., 2018).

Alternative splicing (AS) is a molecular mechanism that produces multiple mRNA transcripts from a single gene locus via the alternative selection of splicing sites during precursor mRNA (pre-mRNA) processing (Lee and Rio, 2015). This increases transcriptome and proteome complexity. AS events can be grouped into five types: exon skipping (ES), alternative 5′ splice sites (A5SS), alternative 3′ splice sites (A3SS), mutually exclusive exons (MXE), and introns retained (IR). IR is the most common AS event in plants, whereas ES is the most prevalent AS event in animals (Chaudhary et al., 2019). AS is an additional regulatory point of gene expression. First, AS events located in the coding DNA sequence (CDS) can introduce premature termination codon (PTC) into mRNA transcripts, which would be degraded through nonsense-mediated decay (NMD) or be translated into truncated proteins (Kalyna et al., 2012). By producing these non-functional transcript isoforms, AS can regulate the expression level of functional transcript isoforms (encoding full-length protein) whether gene promoter strength is affected or not (James et al., 2012). For example, in rice, Fe-deficiency caused AS events in the third intron of *SULFATE TRANSPORTER 3;2* (*OsSultr3;2*) without changing gene expression level, which accelerated the production of isoforms that contain PTC and repressed the expression of the isoform of the functional sulfate transporter protein (Dong et al., 2018). Second, AS events located in the untranslated regions (UTRs) probably affect the localization, stability, and translation efficiency of mRNA, and thus regulate gene expression at the post-transcription level (Pesole et al., 2001; Chatterjee and Pal, 2009; Teramura et al., 2012; Jia et al., 2020). Take rice *OsMac1*, for example, AS events between the first and second exons resulted in the formation of three isoforms of the gene that differed in the structure of 5′ UTR (UTRa, UTRb, and UTRc, respectively). The translation efficiency of UTRc is much higher than that of UTRa and UTRb (Teramura et al., 2012). AS also results in multiple transcript isoforms that encode proteins differing in structure, function, and subcellular location, thus enhancing the diversity of proteome (Brummell et al., 2011; Seo et al., 2011; Kriechbaumer et al., 2012; Vaneechoutte et al., 2017). Some alternate protein isoforms can compete with functional isoforms for the same binding site (Posé et al., 2013). Like transcription regulation, AS is a significant driver of transcriptome reprogramming.

Transcription changes of plants in response to low K^+^ stress had been studied in several species, including rice, maize, and tobacco (Lu et al., 2015; Zhang et al., 2017; Ma et al., 2020). However, there were few studies about the global analysis of AS under low K^+^ stress in plants (Nishida et al., 2017). In this study, we performed a genome-wide analysis of AS in tobacco seedlings subjected to low K^+^ stress, which was compared with changes in expression profiles, to examine the regulatory mechanism of AS and the relationship between AS and transcription regulation under our experimental condition. Our findings suggest that AS modulation and transcription regulation worked independently in response to low K^+^ stress in tobacco. Notably, we found many novel K^+^ deficiency response transcription factors. We also found that auxin response was regulated at AS levels in tobacco roots under the low K^+^ condition.

## Materials and Methods

### Plant Materials and Low K^+^ Treatment

Experiments were performed in a greenhouse under natural illumination and day/night temperatures of 28/22°C. Seeds of tobacco (*Nicotiana tabacum* L. genotypes “Yunyan1” were germinated in trays with peat and vermiculite. 25-days-old seedlings of tobacco were transferred to 1/4 Hoagland’s nutrient solution in which KH_2_PO_4_ and KNO_3_ were replaced by Ca(NO_3_)_2_, and NaH_2_PO_4_ respectively. K^+^ was supplemented by using K_2_SO_4_. Seedlings were exposed to control (2.5 mM) and low K^+^ (0.01 mM) liquid medium for 14 days. The experiment was conducted as a completely randomized design with three biological replicates. The nutrient solution was replaced every 2 days. After the exposure period, roots and shoots were sampled separately.

### Determination of Physiological Changes

Tobacco tissues were dried at 70°C for 3 days and weighted by a precision balance (dry weight). Total root lengths were measured by root analysis machine (WinRhizoV4.0b, Regent Instrument, Canada). Total lateral root (LR) length was computed as total root length minus top root length. A ruler measured LR length, and the LR number was counted by the naked eye. Second-order LR density was calculated by dividing the number of second-order LRs by the lengths of first-order LRs. Indole-3-acetic acid (IAA) concentrations in leaf and root were determined as described by Song et al. (2011).

### RNA Isolation and Library Preparation

Tobacco shoots and roots under control and low K^+^ conditions were collected for RNA-seq analysis. Total RNA was extracted using TRIzol reagent and digested with RNase-free DNase (Qiagen, Germany) according to the manufacturer’s instructions. RNA was then purified and concentrated using an RNeasy column (Takara, Japan). RNA integrity numbers (RINs) was accessed by Agilent 2100 bioanalyzer (Agilent Technologies, United States). The quantification of RNA were assessed using Qubit 4 Fluorometer (Thermo Fisher Scientific Company, Germany). Purification of the poly-A mRNA and construction of the cDNA libraries were performed using the TruSeq RNA sample preparation kit (Illumina, United States). RNA sequencing was performed using the Illumina NovaSeq platform with a standard 150 × 2 bp paired-end sequencing (Zhejiang Annoroad Biotechnology Co., Ltd., China).

### RNA-seq Data Processing and Transcript Assembly

RNA-seq datasets were analyzed as described by Pertea et al. (2016) with custom modification. Raw data were firstly cleaned using Trimmomatic v0.36 (Bolger et al., 2014), with adapters, low-quality bases, and short reads (<40 bp). Cleaned data were then aligned to the *N. tabacum* reference genome^1^ using STAR v2.7.8a (Dobin et al., 2013) with a splicing-aware method and two-pass mode. The genome version of Edwards et al. (2017) was employed in the study (Edwards et al., 2017). The aligned reads were separately assembled into transcripts for each sample with the reference annotation-based transcript (RABT) assembly algorithm and generated an updated transcript annotation with GTF-formatted file using StringTie v2.1.3 (Kovaka et al., 2019). Finally, the expression level of genes was quantified and normalized with the above-updated GTF file using cuffquant and cuffnorm, respectively (Trapnell et al., 2012). Only the genes with an FPKM (fragments per kilobase of transcript per million fragments mapped) >1 in at least two samples were used for downstream genes expression analysis.

### Differential Gene Expression Analysis

DESeq2 was used to perform pairwise comparisons between conditional samples to identify differentially expressed (DE) genes with the updated GTF file (Love et al., 2014). In the study, genes were considered as DE according to the following criteria: (I) Fold change should be >1.5; (II) the adjusted *p*-value from DEseq analyses had to be <0.05.

### Alternative Splicing Events Detection and Differential Alternative Splicing Analysis

Alternative splicing events were identified using rMATS v4.1.1 (Shen S. et al., 2014) with the updated GTF file. Each AS event was supported by two isoforms from an alternatively spliced region. Five types of AS events were then classified, including ES, A5SS, A3SS, MXE, and IR. Differentially alternatively spliced (DAS) events between control and low K^+^ treatment were identified using rMATS with the FDR < 0.05 and ΔPSI ≥ 0.1 (Shen S. et al., 2014). Besides, SUPPA2 (Trincado et al., 2018) and ASprofile (Florea et al., 2013) were also employed for evaluating the reliability of rMATs program.

### Gene Ontology Enrichment Analysis

Genes related to low K^+^ stress were then performed for Gene Ontology (GO) enrichment analysis using agriGO v2.0 toolkit (Tian et al., 2017). Significantly overrepresented GO terms were detected via Fisher’s exact test, and multi-test adjustment was made using Yekutieli (FDR under dependency) method with a cutoff of FDR < 0.05. The overrepresented GO terms were then summarized and visualized with REVIGO web server (Supek et al., 2011).

## Results

### Physiological Responses to Low K^+^+ Stress in Tobacco Seedlings

**FIGURE 1:**
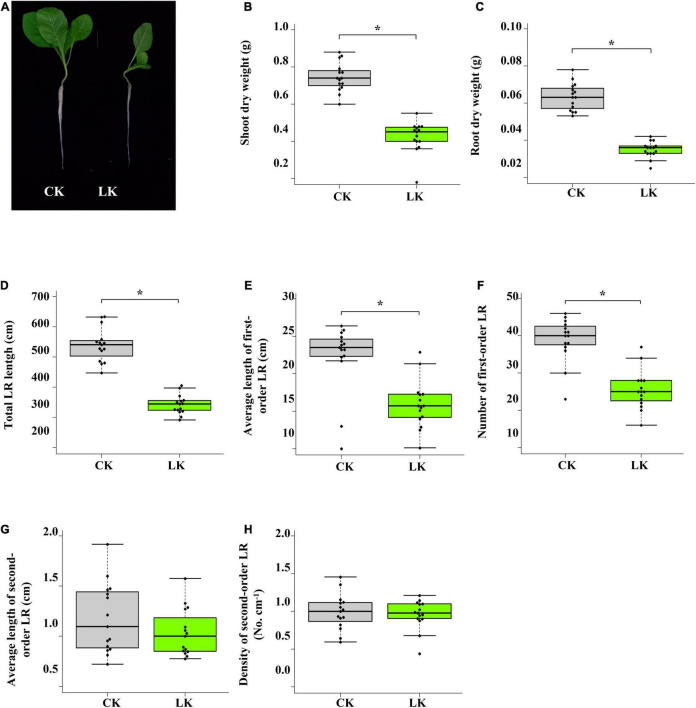
Physiological changes of tobacco seedlings in response to low K^+^ stress. **(A)** Photographs of tobacco seedlings after 14 days of low K^+^ treatment; **(B)** Change of shoot biomass in response to low K^+^ stress; **(C)** Change of root biomass in response to low K^+^ stress. **(D–H)** Root architecture changes in response to low K^+^ stress. Seedlings were subjected to low K^+^ stress (LK, 0.01 mM) or normal nutrition (Control, 2 mM) for 14 days. LR: lateral root. *p < 0.05 (Student’s *t*-test).

The influence of low K^+^ stress on the growth of tobacco seedlings was monitored on the 14th day of treatment (Figure 1). Compared with control seedlings, shoot and root biomass of K^+^-deficient seedlings reduced nearly by half (Figures 1B,C). Besides, root system architecture was dramatically changed due to K^+^ deficiency (Figures 1D–H). Total LR length, the number of first-order LR, and an average length of first-order LR were significantly reduced under low K^+^ stress, while density and an average second-order LR were seldom affected. However, the leaf number of K^+^-deficient seedlings was the same as that of control seedlings (Figure 1A). These results showed that the growth of tobacco seedlings was inhibited by low K^+^ stress, whereas K^+^-deficient seedlings and control seedlings were in the same development stage.

### Transcript Assembly and Gene Prediction

RNA-seq data was collected from shoots and roots in both low K^+^ and control conditions. A total of 11 RNA-seq samples were examined herein, which were divided into four groups, control root (CKR), low K^+^ root (LKR), control shoot (CKS), and low K^+^ shoot (LKS) (Supplementary Table 1). Cleaned data (∼30 Gb) was first mapped to the tobacco reference genome, and then alignments were assembled into transcripts in each sample. Finally, transcripts from all samples were merged to form a unified set of transcripts, which consisted of 55,175 genes and 113,637 transcripts. Of the predicted genes, 62.6% (34,560) had only one transcript, and 37.4% (20,615) had two or more transcripts. Moreover, 32.0% (17,659) of the genes had only one exon, and 68.0% (37,516) had two or more exons. The average number of transcripts per gene is 2.059574, and the average number of exons per transcript is 5.634679. There were 38,330 genes with FPKM more than 1 in at least two samples.

### Occurrence of Alternative Splicing Events in Tobacco Seedling

**FIGURE 2:**
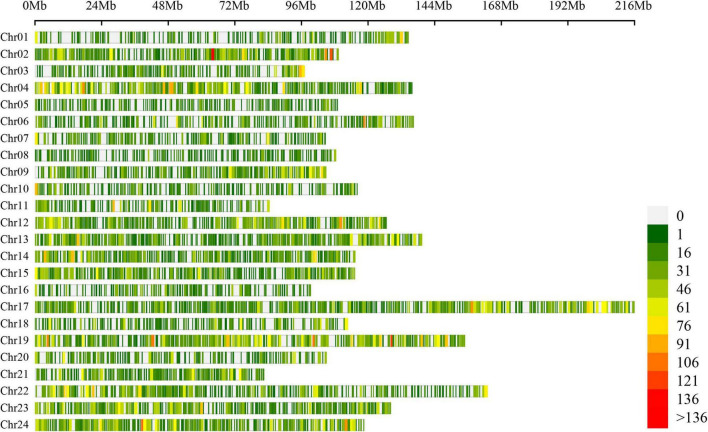
Chromosome distribution of AS events in tobacco genome. Figures on the right hand of the color bar indicated the number of AS events within the 1 Mb window size. Chr: chromosome.

**FIGURE 3:**
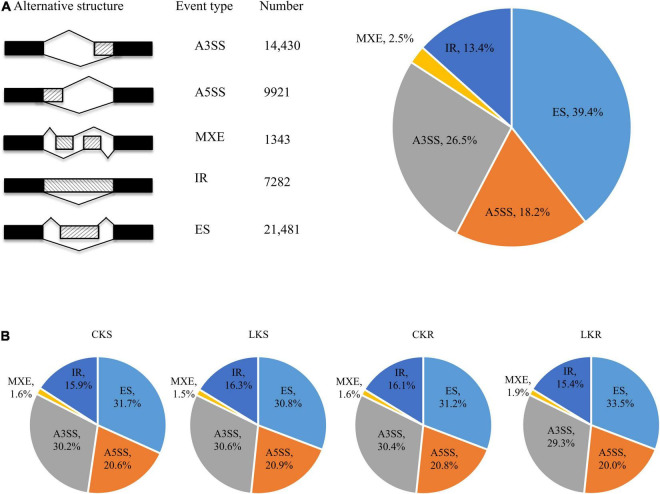
Distribution of AS types. **(A)** Five different types of AS events and their frequency. The first column illustrates the structure of the AS events, followed by its name, the number, and the percentage of events; **(B)** The distribution of AS types in CKS, LKS, CKR, and LKR. CKS: shoot in control condition; LKS: shoot in low K^+^ stress; CKR: root in control condition; LKR: root in low K^+^ stress.

**FIGURE 4:**
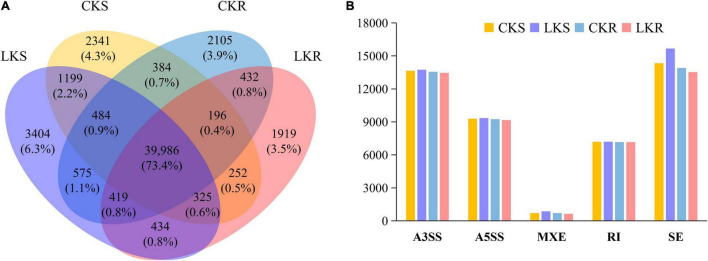
Response of AS events to low K^+^ stress in shoots and roots. **(A)** Venn diagram of AS events in shoots and roots at the control and low K^+^ stress conditions; **(B)** The number of AS events in shoots and roots at the control and low K^+^ stress conditions. CKS: shoot in control condition; LKS: shoot in low K^+^ stress; CKR: root in control condition; LKR: root in low K^+^ stress.

A total of 54,457 AS events from 28,179 genes were identified in all samples, implying that AS events occurred in multi-exonic genes. Besides, 39,370 AS events were detected from known annotated and 15,087 from novel events. These AS events were extensively distributed in the tobacco genome (Figure 2). Among AS types, ES was the most abundant (39.4%) type of AS events, followed by A3SS (26.5%), A5SS (18.2%), and IR (13.4%), with MXE constituting as low as 2.5% of total AS events (Figure 3A). The distribution of AS types in tobacco differed from the previous reports in wheat, *Arabidopsis*, and rice (Calixto et al., 2018; Dong et al., 2018; Liu et al., 2018), in which IR was the most common type of AS events, but agreed with the report in tea (Ding et al., 2020) in which IR was less common than ES, A3SS, and A5SS. SUPPA2 and ASprofile programs were further employed for evaluating whether the percentage of IR type was underestimated by rMATs algorithm. As a result, RI (also named IR) was 14% in SUPPA2 (Supplementary Figure 1) and IR was only 6% in ASprofile (Supplementary Figure 2). Overall, the different data analysis tools performed similar results and rMATs showed good performance as described by Mehmood et al. (2020). The ratios of five AS types in CKR, LKR, CKS, and LKS were consistent with total events (Figure 3B). Besides, 39,984 events (73.4% of total events) were present in all four groups (Figure 4A), with 4830 (8.9%) and 5757 (10.6%) events were specific to control and low K^+^ stress conditions. The number of AS events were seldom affected by low K^+^ stress except for ES event, whose number increased in shoot and decreased in root under stress (Figure 4B).

### Identification of Differentially Alternatively Spliced Events in Response to Low K^+^+ Stress

**FIGURE 5:**
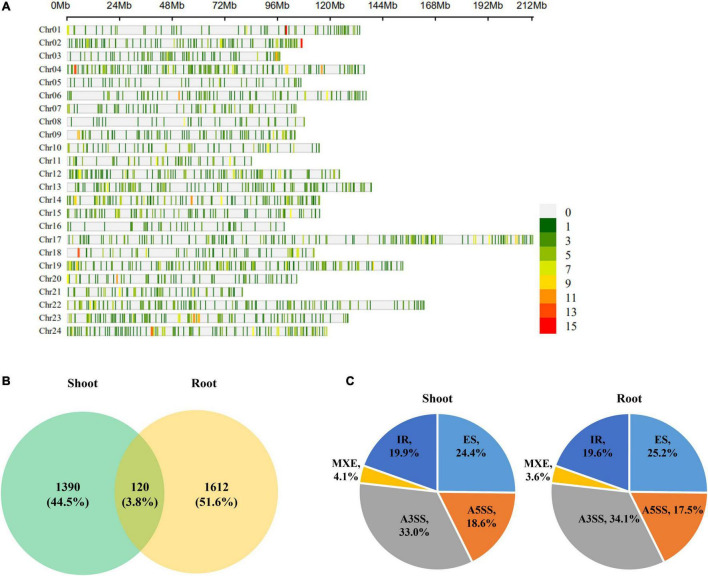
Differentially alternatively spliced (DAS) events analyses of tobacco in response to low K^+^ stress. **(A)** Chromosome distribution of DAS events in tobacco genome. The color bar on the right hand indicated the number of AS events within 1 Mb window size; **(B)** Venn diagram of DAS events in response to low K^+^ stress in shoots and roots; **(C)** The distribution of different types of DAS events in shoots and roots. Chr: chromosome.

To identify AS events that were significantly sensitive to K^+^ deficiency, DAS analysis under low K^+^ stress was performed using rMATS (Shen S. et al., 2014) software. Total 3,122 DAS events were identified, referring to 2,120 genes. These DAS events are sporadically scattered in the tobacco genome (Figure 5A). 1,510 and 1,732 DAS events were identified in shoots and roots under low K^+^ stress, involving 1,152 and 1,253 genes, respectively. Only 120 DAS events occurred in both shoots and roots, which occupied 3.8% of total DAS events (3,122), implying that most DAS events were tissue-specific (Figure 5B). The distribution of different DAS events in the shoot was identical to that in roots (Figure 5C). A3SS became dominant in five AS events, occupying about one-third of DAS events. ES occupied one four of total DAS events, followed by IR and A5SS, which occupied nearly one-fifth of DAS events, respectively. MXE, the rarest AS type, occupied about 4% of DAS events.

### Association Analysis of Differentially Expressed Genes and Differentially Alternatively Spliced Genes Under Low K^+^+ Stress

**FIGURE 6:**
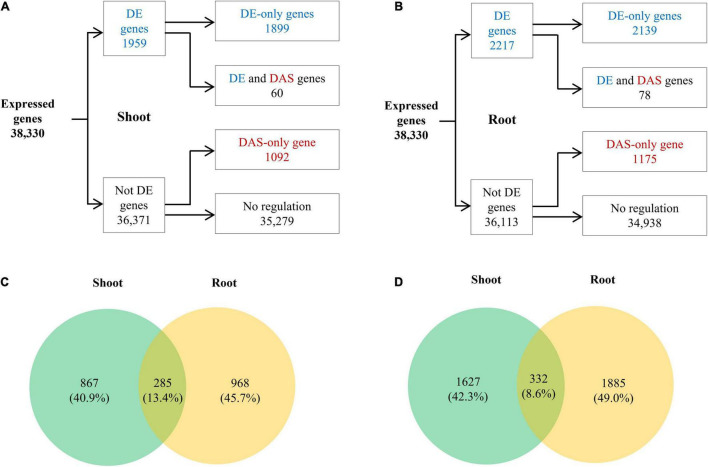
Differentially expressed (DE) and DAS analyses of tobacco in response to low K^+^ stress. **(A)** Flow chart showing the distribution of the DE (blue) and DAS (red) genes in shoots; **(B)** Flow chart showing the distribution of the DE (blue) and DAS (red) genes in roots; **(C)** Venn diagram of DAS genes in shoots and roots; **(D)** Venn diagram of DE genes in shoots and roots.

We further explored the relationship between transcription regulation and AS modulation under low K^+^ by a comparative analysis of DE and DAS genes. For both shoots and roots, there were more DE genes than DAS genes. 1,959 DE genes and 1,152 DAS genes were identified in shoots, 60 of which were regulated by both transcription and AS (Figure 6A). The proportion of DAS genes in DE genes was equal to that in non-DE genes (Supplementary Figure 3). Meanwhile, 2,217 DE and 1,253 DAS genes were identified in roots, with 78 genes regulated at both transcription and AS levels (Figure 6B). The proportion of DAS genes in DE genes was equal to that in non-DE genes (Supplementary Figure 3). In addition, 285 genes (13.4% of total DAS genes) were DAS in both shoots and roots (Figure 6C), with 332 genes (8.6% of total DE genes) DE in both the two tissues (Figure 6D), suggesting that both DAS genes and DE genes were tissue-specific. Notably, *HAK5*, the marker gene for low K^+^ response (Gierth et al., 2005; Wang et al., 2021), was significantly induced in root under K^+^ deficiency (Supplementary Figure 4), implying K^+^ deficiency response was induced under our experiment condition.

### Gene Ontology Enrichment Analysis of Differentially Expressed Genes and Differentially Alternatively Spliced Genes

**FIGURE 7:**
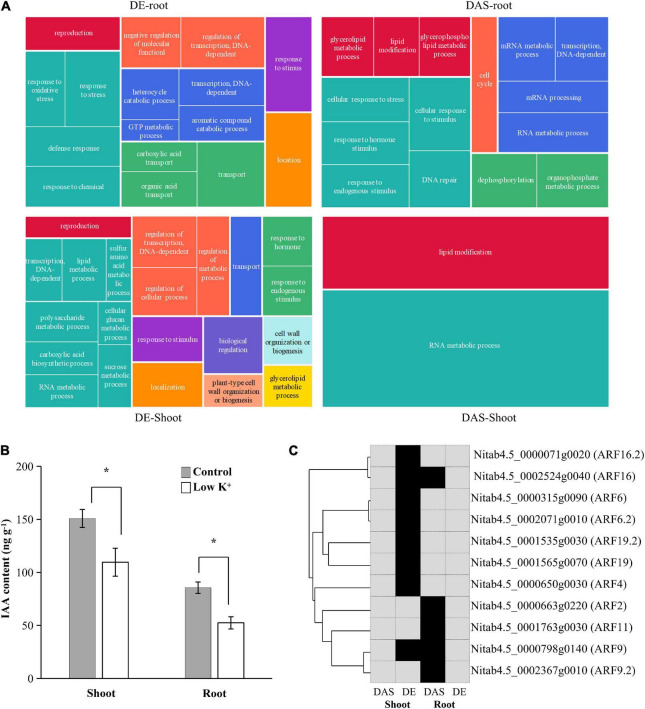
Gene Ontology (GO) enrichment analysis of DAS and DE genes in shoots and roots. **(A)** Tree maps of representative GO terms in DAS and DE genes in shoots and roots. The drawings were plotted using REVIGO; **(B)** IAA content in leaves and roots of tobacco seedlings under control and low K^+^ conditions. *p < 0.05 student’s *t*-test; **(C)** ARF transcription factors involved in GO term “Response to hormone.” The phylogenetic relationship of ARF transcription factors is shown on the left. Black, genes were differentially expressed (DE) or differentially alternatively spliced (DAS); Gray, genes were no significant difference.

Since DE and DAS gene sets were vastly different from each other, and both were tissue-specific, we examined the overrepresented GO terms for shoot DE genes, shoot DAS genes, root DE genes, and root DAS genes separately (Figure 7A and Supplementary Table 2). In the shoot, GO terms such as “regulation of transcription, DNA-dependent” (GO:0006355), “transport” (GO:0006810), “reproduction” (GO:0000003), “response to stimulus” (GO:0050896), and “cell wall organization or biogenesis” (GO:0071554) as well as some terms related to metabolic processes of polysaccharide, lipid, carboxylic acid, and amino acid were specific to DE genes. In contrast, the GO term “lipid modification” (GO:0030258) was specific to DAS genes. Only GO terms “RNA metabolic process” (GO:0016070) were overrepresented in DE and DAS genes. In the root, few GO term was overrepresented in both DE and DAS genes, except “transcription, DNA-dependent” (GO:0006351). GO terms that specific to DE genes were mainly related to oxidation-reduction, transcription regulation, transport, and response to stress, whereas GO terms that specific to DAS genes were mainly related to RNA metabolism, lipid modification, and DNA repair. The GO terms differed significantly between DE and DAS genes in both of the two tissues.

### Differentially Expressed and Differentially Alternatively Spliced Analyses Identify K^+^ Deficiency Response Transcription Factors

With RNA-seq data, we found 175 K^+^ deficiency response transcription factors (Supplementary Table 3 and Supplementary Figure 5). Among them, 142 transcription factors were DE, of which 80 and 75 were identified from shoot and root, respectively. Besides, 45 transcription factors were differentially alternative spliced, of which 21 and 30 were identified from shoot and root, respectively. For DE transcription factors, WRKY transcription factors were the most frequent, followed by HD-ZIP, bHLH, and NAC family. For DAS transcription factors, the top three transcription factor families were bHLH, ARF, and bZIP. Twelve transcription factors were regulated at both expression abundance change and AS pattern, belonging to six transcription factor families (Supplementary Table 3).

### Role of Alternative Splicing in Response to Auxin

Since ARF transcription factors played an essential role in auxin signaling transduction, changes in expression of *ARF* genes indicated that auxin content in seedlings had changed. Thus, we measured IAA contents of leaves and roots under control and low K^+^ conditions (Figure 7B). Compared with control plants, IAA content in leaf and root of K^+^-deficient plants declined by about 30 and 40%, respectively. Auxin concentration levels decreased in leaves and roots subjected to low K^+^ stress, which was consistent with previous studies (Song et al., 2015).

The GO term “Response to hormone stimulus” (GO:0009725) was significantly overrepresented in both root DAS genes and shoot DE genes since five ARF transcription factors were DAS in root and eight ARF transcription factors were DE in shoots. The five DAS ARF transcription factors were not DE in root, while the eight DE ARF transcription factors were not DAS in the shoot (Figure 7C). The auxin response was subjected to transcription regulation in shoot and to AS modulation in the root.

**FIGURE 8:**
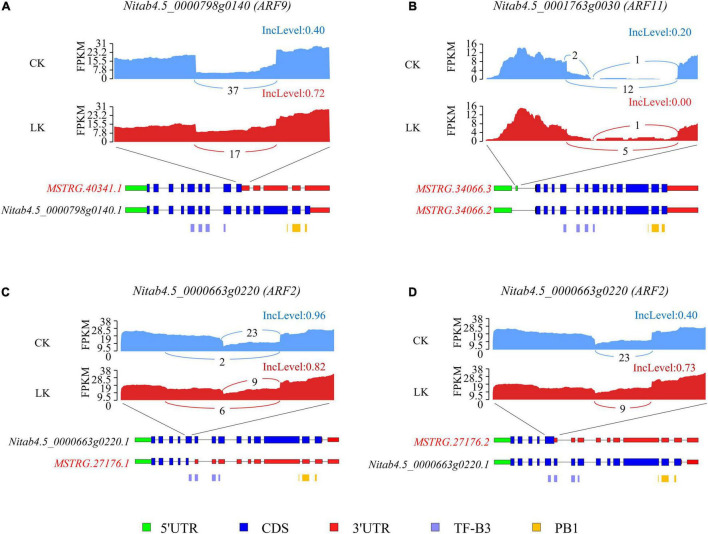
Alternative splicing (AS) of three ARF genes under K^+^ deficiency conditions in tobacco root. Visualization of the exon-intron structure of representative transcripts for ARF9 **(A)**, ARF11 **(B)**, and ARF2 **(C,D)** genes in root under K^+^ deficiency conditions. Sashimi plots show the number of RNA-seq reads mapping to loci associated with AS events. The heights of the bars represent FPKM. Structure diagrams below the sashimi plots show the 5′ UTRs (green), coding exons (blue), 3′ UTRs (red), and specific protein domains (purple, orange) in major transcript isoforms. Upper numbers in the sashimi plots indicate the inclusion counts (IJC), and lower numbers indicate the skipping counts (SJC) described in Figure 3A. CK: control condition; LK: low K^+^ stress; IncLevel: inclusion level.

Further, we evaluated the consequence of DAS events on ARF transcription factors. Tobacco ARF transcription factors contained an amino-terminal DNA-binding domain (TF-B3) and a carboxy-terminal dimerization domain (PB1). For *ARF9* (*Nitab4.5_0000798g0140*), K^+^ deficiency significantly induced the ratio of IR of the ninth intron, which introduced a PTC and may lead to the removal of the region encoding the PB1 domain or NMD. Under control conditions, about 40% of reads retained the ninth intron, while under low K^+^ stress, the figure incensed sharply to 72% (Figure 8A). As a result, the proportion of the functional isoform (*Nitab4.5_0000798g0140.1*) was reduced by half due to K^+^ deficiency. Furthermore, an ES event was identified in the 5′ UTR region of *ARF11* (*Nitab4.5_0001763g0030*). Whether the exon was skipped or not, the CDS region was not affected (Figure 8B). However, this event may affect the stability or translation efficiency of mRNA of *ARF11* (Jia et al., 2020). For *ARF2* (*Nitab4.5_0000663g0220*), K^+^ deficiency led to more isoforms with truncated exon 5 (Figure 8C) and increased the ratio of IR of intron 5 (Figure 8D). Both truncated exon 5 and retained intron 5 introduced PTC, resulting in truncated proteins or causing NMD. As a result, the ratio of a functional isoform of *ARF2* declined due to K^+^ deficiency.

## Discussion

### K^+^+ Deficiency Significantly Affected the Growth of Tobacco Seedlings

We first determined the appropriate experiment condition of low K^+^ treatment to investigate the transcriptome changes in tobacco seedlings under K^+^ deficiency. Uniform seedlings (25-days old) were transferred to low K^+^ (0.01 mM) and control (1 mM) conditions. After 14 days, tobacco seedlings showed significant character differences between two different K^+^ environments. K^+^-deficient seedlings had smaller shoots and roots than those of control seedlings (Figure 1A). Compared with control seedlings, the biomass of shoots and roots of K^+^-deficient seedlings was significantly lower (Figures 1B,C). Under K^+^ deficiency, total LR root length was significantly reduced, attributed to reduced root length and root number of first-order LR (Figures 1D–H). These results showed that K^+^ deficiency significantly affected the growth of tobacco seedlings. However, the leaf number of control seedlings and K^+^-deficient seedlings was the same, indicating that K^+^-deficient seedlings and control seedlings were in the same development stage (Figure 1A). Therefore the differences in transcriptome between control and K^+^-deficient samples mainly came from low K^+^ stress rather than two different development stage.

### Characters of Alternative Splicing Events in Tobacco

This study identified 54,457 AS events from 28,179 genes in tobacco shoots and roots, which accounted for 51.1% of total genes. This ratio was higher in tobacco than in many other plants, like maize (40%), *Arabidopsis* (49%), soybean (63%), and cotton (32%) (Ding et al., 2014; Shen Y. et al., 2014; Thatcher et al., 2014; Zhu et al., 2018), suggesting that AS frequency varied across species. AS events were extensively distributed in the tobacco genome (Figure 2), showing no position bias. ES was the most abundant AS type in tobacco (Figure 3), though IR was the most abundant in many other species (Calixto et al., 2018; Dong et al., 2018; Liu et al., 2018), which means that IR was not always the most common event type in the plant. SUPPA2 and ASprofile programs were employed and also found a close percentage of IR type. Besides, most AS events (73.4%) were commonly identified in all four tissue/treatment conditions. Only 8.9 and 10.6% of total AS events were specific to control and low K^+^ conditions, respectively (Figure 4A). Whether in shoots or roots, the number of AS events were not induced by low K^+^ stress except ES event, which was increased in shoot and decreased in roots (Figure 4B). Unlike cotton and *Arabidopsis* (Ding et al., 2014; Zhu et al., 2018), AS was not significantly enhanced by stress in tobacco.

Although AS was not obviously induced by low K^+^ stress, 3122 DAS events were identified from tobacco seedlings, of which 1510 and 1732 were identified in shoots and roots, respectively. They sporadically scattered among the genome, showing no distinct position bias (Figure 5A). Most DAS events were tissue-specific, and only 120 DAS events were identified in both shoot and root (Figure 5B). DAS genes were slightly less in the shoot (1152) than in root (1253), whereas the ratios of DAS event to DAS gene were almost the same in the two issues (1.31 in shoot vs. 1.38 in root). Both in shoot and root, A3SS overtaken ES as the most frequent DAS events. Moreover, the distribution of different DAS events in shoot and root was the same (Figure 5C). These results suggested that low K^+^ stress affected gene expression by inducing DAS rather than increasing the frequency of AS events.

### Alternative Splicing Modulation and Transcription Regulation Worked Independently Under Low K^+^ Stress

Previous studies have shown that AS modulation and transcription regulation regulated gene expression in response to environmental stress (Staiger and Brown, 2013; Calixto et al., 2018; Laloum et al., 2018), but questions and disputes appeared regarding the association relationship between transcription regulation and AS modulation. The main focus is whether AS and transcription regulation work independently or associate with each other under stress. It had been shown that the association relationship between transcription and AS regulation change with different species, different stresses, and different processing times. In rice, DE genes and DAS genes displayed little overlap under mineral (Fe, Zn, Cu, Mn, and P) deficiency, whereas in the tea plant, DAS genes significantly overlapped with DE genes under drought stress (Dong et al., 2018; Ding et al., 2020). In wheat, the situation was even more complicated. At 1 h of heat treatment, transcription regulation was significantly associated with AS modulation, but at 6 h, they were uncorrelated (Liu et al., 2018). However, there was no study about the relationship between AS modulation and transcription regulation under low K^+^ stress in tobacco.

In our study, 1959 and 2217 DE genes were identified in shoot and roots, respectively. Like DAS genes, DE genes were slightly less in shoot than in root, which was not surprising because the root was the primary organ that senses environmental K^+^ concentration and absorbs K^+^. Both in shoot and the root, the proportion of DAS genes in DE genes equaled that in non-DE genes (Supplementary Figure 3). GO analysis (Figure 7A and Supplementary Table 2) showed that GO terms overrepresented in DE genes mainly were related to transcription regulation, transcription, oxidation-reduction, transport, response to stimulus, cell wall organization or biogenesis, and various metabolic processes. These results were consistent with the previous studies in rice, maize, and tobacco (Lu et al., 2015; Zhang et al., 2017; Ma et al., 2020). However, GO terms in DAS genes differed significantly from DE genes, mainly associated with mRNA processing, DNA repair, lipid metabolism, and DNA repair. Meanwhile, a few GO terms were overlapped between DE and DAS genes. Thus transcription regulation and AS modulation worked independently in response to low K^+^ stress in tobacco, and their target biological processes were different.

### Transcription Factors Responsive to Low K^+^ Stress

We identified 142 DE transcription factors and 45 DAS transcription factors from tobacco seedlings under low K^+^ stress (Supplementary Table 3 and Supplementary Figure 5). Only 12 DE transcription factors were regulated at AS levels, indicating that transcription regulation and AS modulation regulated different biological processes. The 45 DAS transcription factors belonged to 18 families, including bHLH, ARF, bZIP, etc. None of them were regulated at AS level under low K^+^ stress. Lu et al. (2015) had identified 57 DE transcription factors from tobacco root within 24 h of low K^+^ treatment using microarray data. However, there was little overlap between the K^+^ deficiency response DE transcription factors identified in the previous study and the DE transcription factors identified here, probably because of the different experimental conditions. Thus nearly 200 novel K^+^ deficiency response transcription factors were identified in our study. The research on the function of these transcription factors will be helpful to clarify the molecular mechanism underlying the K^+^ deficiency response.

### ARF Transcription Factors Were Subjected to Alternative Splicing Modulation in Root Under Low K^+^ Stress

Auxin is the first discovered hormone for the plant. It controls various developmental processes and plays an essential role in plant growth and morphogenesis, particularly root development (Friml, 2003; De Smet et al., 2006). Unlike the model plant *Arabidopsis* and rice, tobacco’s root system is mainly composed of the LRs, with the taproot being relatively underdeveloped. Previous studies showed that auxin was involved in LR formation under low K^+^ stress and drought stress in tobacco seedlings (Song et al., 2015; Wang et al., 2018). ARF transcription factors sense auxin signaling and then regulate the expression of auxin response genes (Guilfoyle and Hagen, 2007). Most ARFs contain an amino-terminal DNA-binding domain and a carboxy-terminal dimerization domain. In tobacco, the DNA-binding and dimerization domains are described as TF-B3 domain and PB1 domain, respectively.

In our study, IAA contents of leaves and roots were significantly reduced by low K^+^ stress, and the root system showed the classic symptom of auxin deficiency (Figures 1, 7B). In the shoot, DE genes were enriched in GO term “Response to hormone” and eight ARF transcription factors were DE (Figure 7 and Supplementary Tables 2, 3), indicating that response to auxin deficiency was mainly subject to transcription regulation in the shoot. However, root DE genes were not enriched in any GO term related to auxin response, and hardly any ARF was DE in the root. Instead, root DAS genes were enriched in GO term “Response to hormone” and five ARF transcription factors were differently alternatively spliced in the root (Figure 7 and Supplementary Tables 2, 3). These results suggested that response to auxin deficiency was mainly subject to AS regulation in the root.

Further, we examined the DAS pattern of the five AS genes in K^+^-starved root and found two regulation methods: introducing PTC into transcripts and changing the translation efficiency of transcripts. For *Nitab4.5_0000798g0140* (*ARF9*), *Nitab4.5_0002367g0010* (*ARF9.2*), and *Nitab4.5_0000663g0220* (*ARF2*), AS modulation increased the production of isoforms that contain PTC and repressed the expression of the functional isoforms (Figures 8A,C,D and Supplementary Figure 6B). For *Nitab4.5_0001763g0030* (*ARF11*) and *Nitab4.5_0002524g0040.1* (*ARF16*), DAS events were located upstream of the transcription initiation codon, which resulted in the formation of isoforms with different 5′ UTR (Figure 8B and Supplementary Figure 6A). Since the structure of 5′ UTR may affect the translation efficiency of transcripts (Teramura et al., 2012), the two ARF genes were probably regulated at the translation level.

*Arabidopsis AtARF2* is a well-studied transcription factor, which negatively regulated the expression of *HAK5* in response to low K^+^ stress (Zhao et al., 2016; Santa-María et al., 2018). However, previous studies showed that the transcription of the gene is not affected by low K^+^ stress. It is speculated that low K^+^ treatment leads to *AtARF2* phosphorylation and changes its DNA binding activity to the *HAK5* promoter. In our study, the tobacco ortholog of *AtARF2* was not DE under low K^+^ stress either, but it was regulated at AS level in the root. Two DAS events occurred in the CDS region of tobacco *ARF2*, which significantly reduced the ratio of the functional isoform of the gene (Figures 8C,D). These results indicated that *AtARF2* was probably subject to AS regulation in *Arabidopsis* and that tobacco *ARF2* may participate in regulating K^+^ uptake under low K^+^ stress. It will be interesting to study the function of the novel K^+^ deficiency response transcription factors and the function of AS on the known K^+^ deficiency response transcription factors in future experiments.

## Data Availability Statement

The original contributions presented in the study are publicly available. This data can be found here: National Center for Biotechnology Information (NCBI) BioProject database under accession number PRJNA744321.

## Author Contributions

WS and YL conceived and designed the experiments and revised the manuscript. BH and LM performed the experiments and wrote the manuscript. BH, LT, WQ, and FH participated in data collection and analysis. All authors have read and approved the final manuscript.

## Conflict of Interest

The authors declare that the research was conducted in the absence of any commercial or financial relationships that could be construed as a potential conflict of interest.

## Publisher’s Note

All claims expressed in this article are solely those of the authors and do not necessarily represent those of their affiliated organizations, or those of the publisher, the editors and the reviewers. Any product that may be evaluated in this article, or claim that may be made by its manufacturer, is not guaranteed or endorsed by the publisher.

## Footnotes

1
https://solgenomics.net/organism/Nicotiana_tabacum/genome

## Supplementary Material

Supplementary Figure 1 SUPPA2 prediction results of AS events. Different combine sets including different tissues (Root and Shoot), conditions (CK and treatment), and total samples were separately generated.

Supplementary Figure 2 ASprofile prediction results of AS events. Different combine sets including different tissues (Root and Shoot), conditions (CK and treatment), and total samples were separately generated.

Supplementary Figure 3 The proportion of DAS genes in DE genes and non-DE genes in shoot and root under low K^+^ stress condition.

Supplementary Figure 4 Expression level of HAK5 in shoot and root of tobacco seedlings under control and low K^+^ conditions.

Supplementary Figure 5 The distribution of DAS and DE transcription factors among gene families.

Supplementary Figure 6 AS of two ARF genes under K^+^ deficiency conditions in tobacco root. Visualization of the exon-intron structure of representative transcripts for ARF16 **(A)** and ARF9.2 **(B)** genes in root under K^+^ deficiency conditions. Sashimi plots show the number of RNA-seq reads mapping to loci associated with AS events. The heights of the bars represent RPKM. Structure diagrams below the sashimi plots show the 5′ UTRs (green), coding exons (blue), 3′ UTRs (red), and specific protein domains (purple, orange) in major transcript isoforms. Upper numbers in the sashimi plots indicate the inclusion counts (IJC) and lower numbers indicate the skipping counts (SJC) as described in Figure 3A. CK: control condition; LK: low K^+^ stress; IncLevel: inclusion level.

Supplementary Table 1 Summary of RNA-Seq results.

Supplementary Table 2 GO enrichment results of DAS and DE genes in shoot and root.

Supplementary Table 3 Transcription factors in response to low K^+^ in tobacco.
