# Supplementary figures and images for: The Landscape of Alternative Splicing Regulating Potassium Use Efficiency in Nicotiana tabacum
Source: Front Plant Sci. 2021 Nov 8;12:774829. doi: 10.3389/fpls.2021.774829 (PMC8630638; doi:10.3389/fpls.2021.774829)

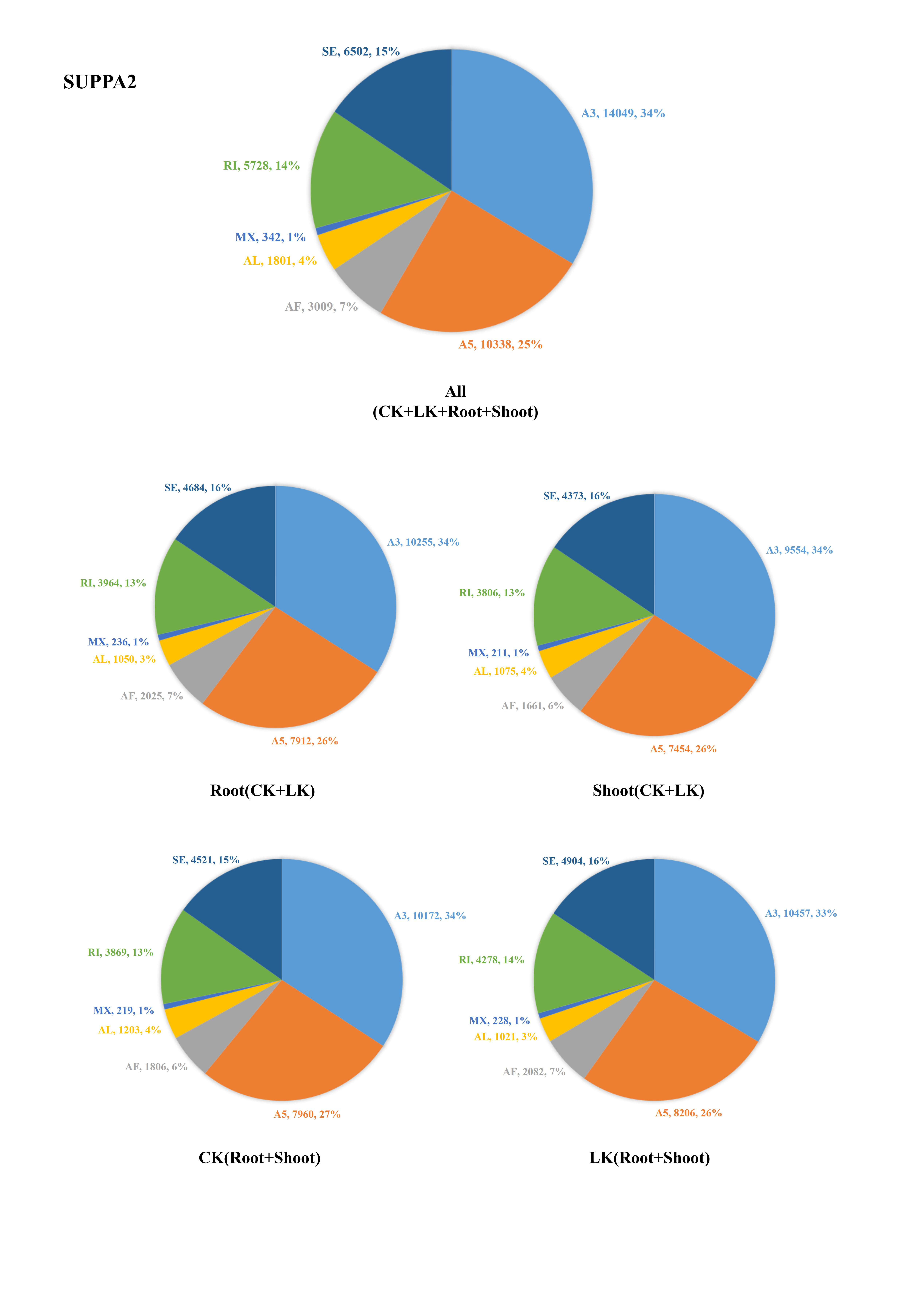

Supplement: Supplementary Figure 1 — SUPPA2 prediction results of AS events. Different combine sets including different tissues (Root and Shoot), conditions (CK and treatment), and total samples were separately generated. [file Image_1.TIF]

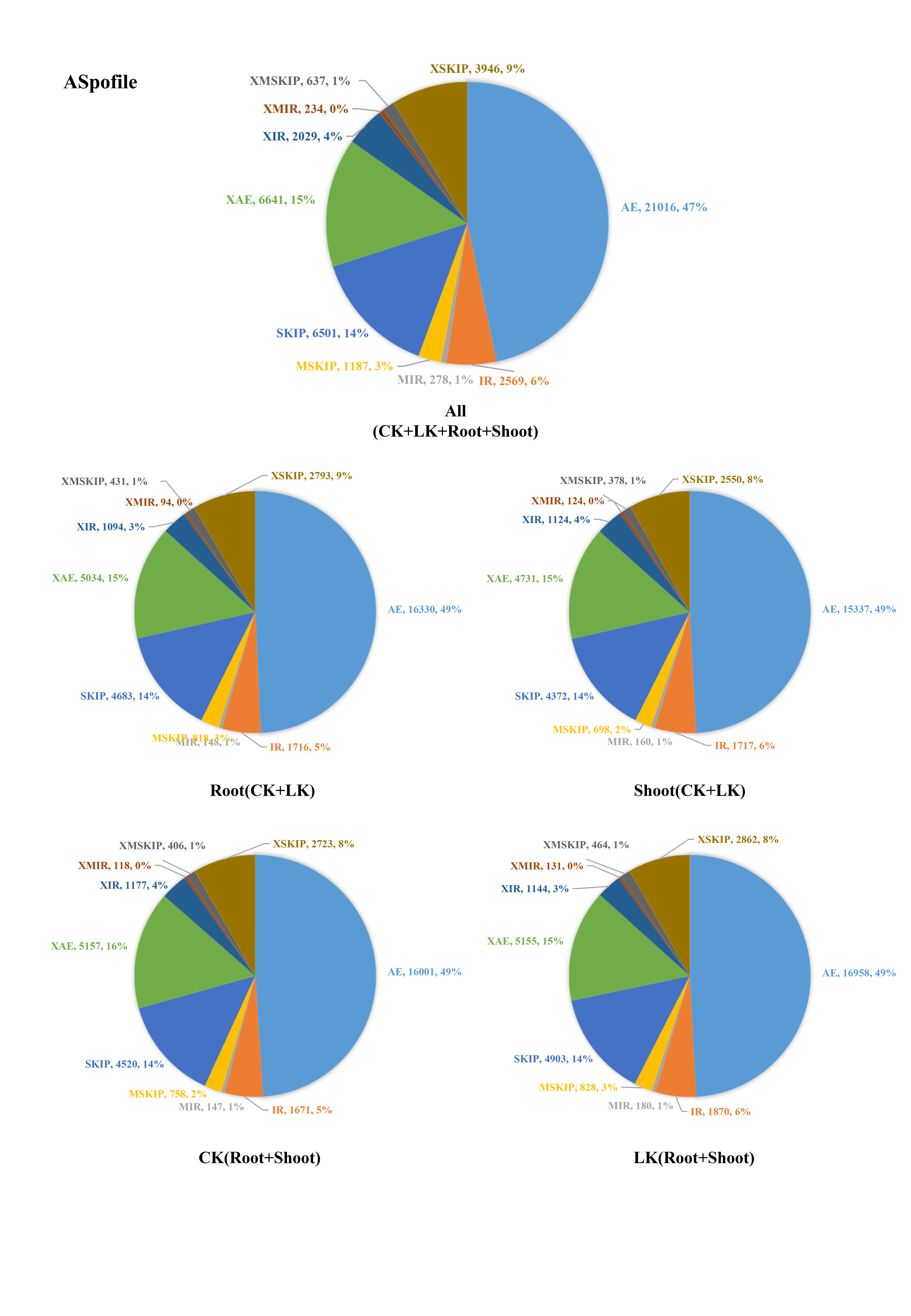

Supplement: Supplementary Figure 2 — ASprofile prediction results of AS events. Different combine sets including different tissues (Root and Shoot), conditions (CK and treatment), and total samples were separately generated. [file Image_2.TIF]

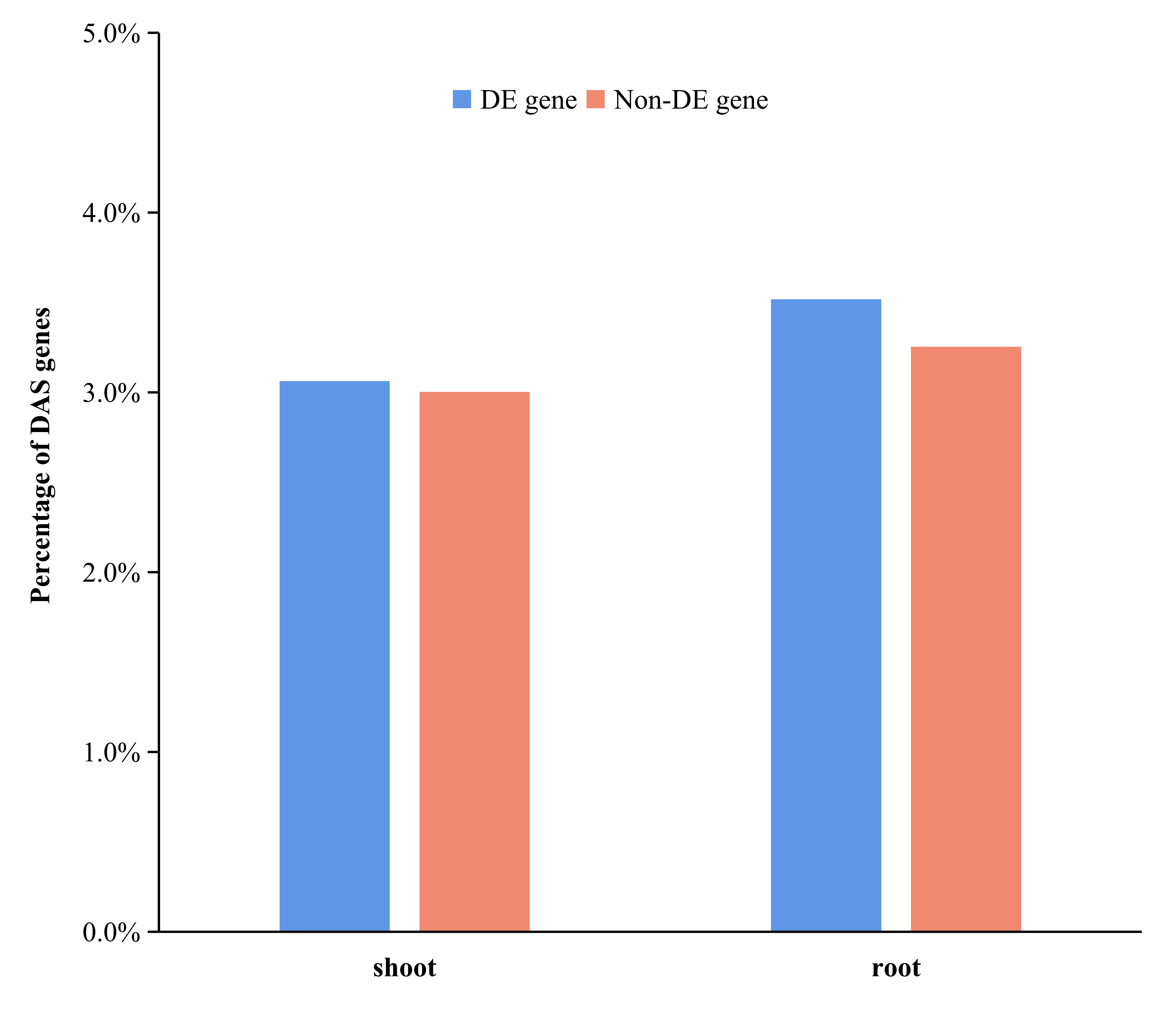

Supplement: Supplementary Figure 3 — The proportion of DAS genes in DE genes and non-DE genes in shoot and root under low K+ stress condition. [file Image_3.TIF]

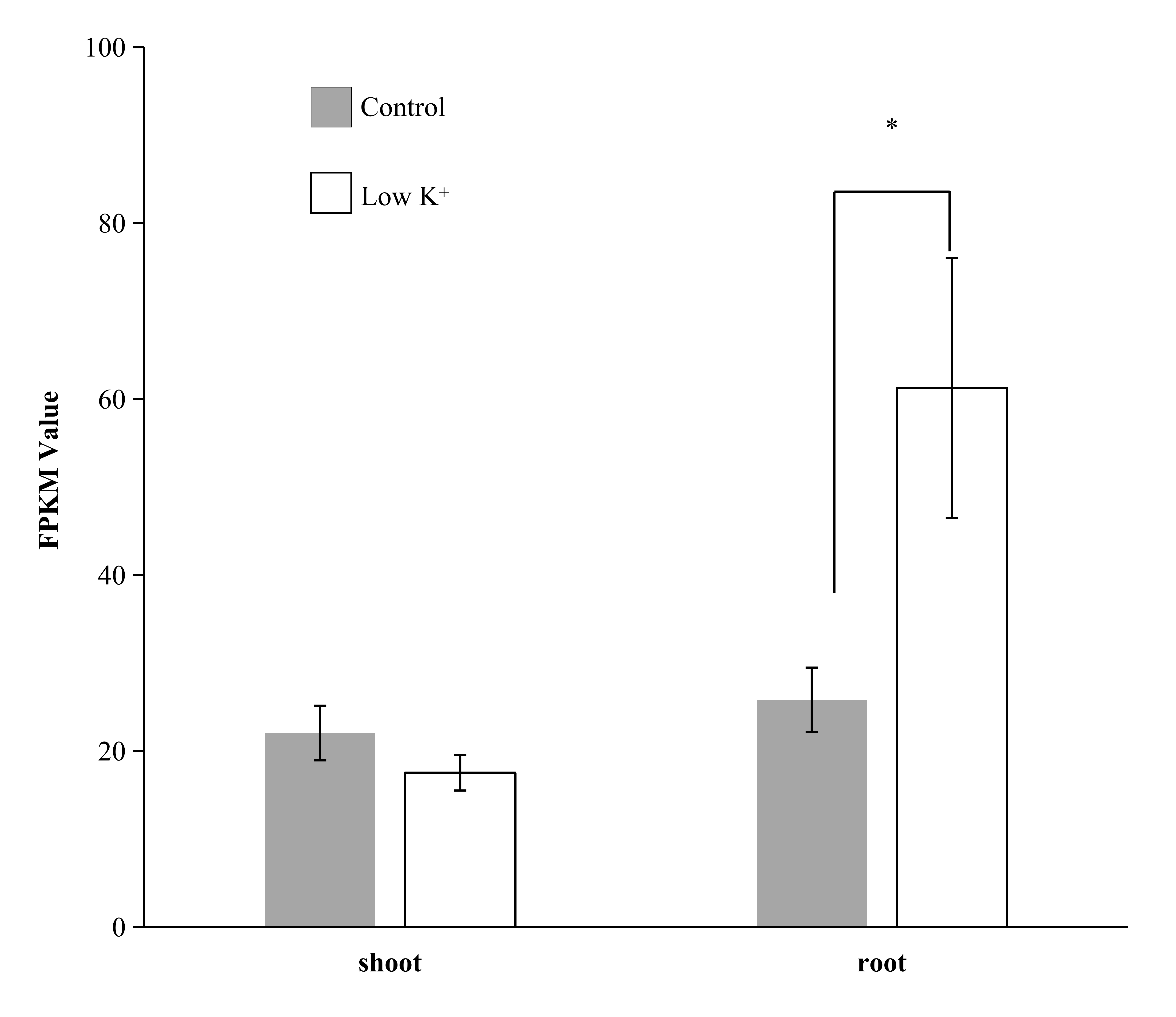

Supplement: Supplementary Figure 4 — Expression level of HAK5 in shoot and root of tobacco seedlings under control and low K+ conditions. [file Image_4.TIF]

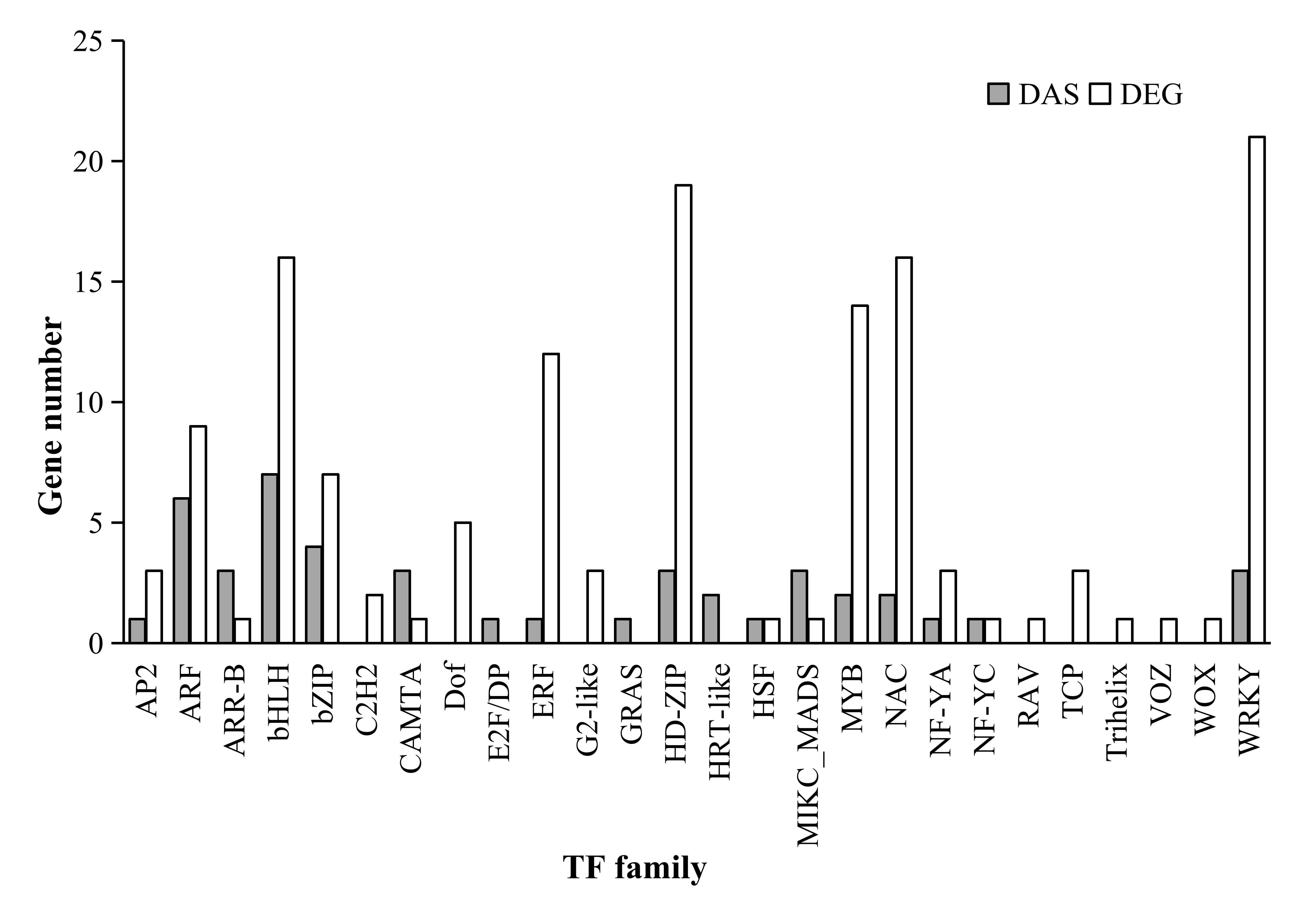

Supplement: Supplementary Figure 5 — The distribution of DAS and DE transcription factors among gene families. [file Image_5.TIF]

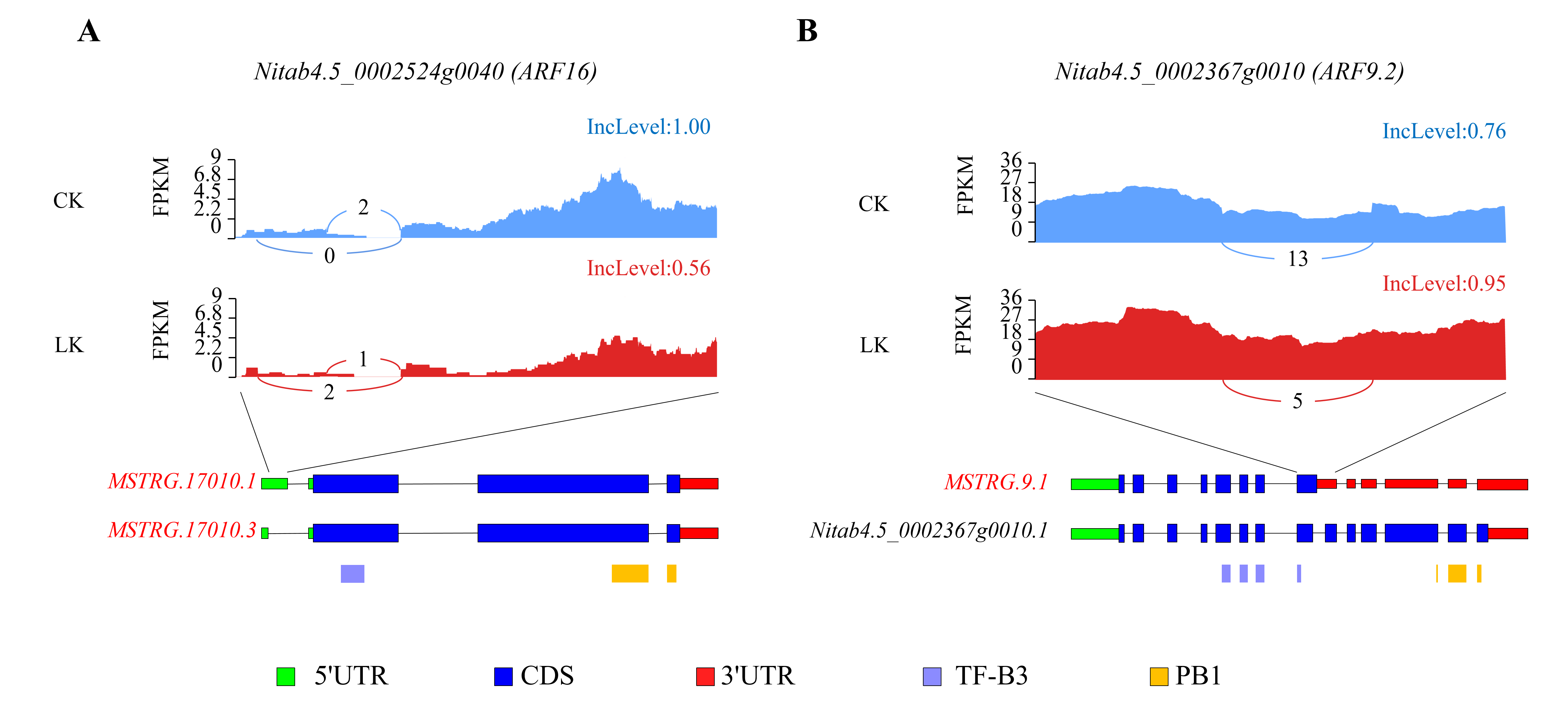

Supplement: Supplementary Figure 6 — AS of two ARF genes under K+ deficiency conditions in tobacco root. Visualization of the exon-intron structure of representative transcripts for ARF16 (A) and ARF9.2 (B) genes in root under K+ deficiency conditions. Sashimi plots show the number of RNA-seq reads mapping to loci associated with AS events. The heights of the bars represent RPKM. Structure diagrams below the sashimi plots show the 5′ UTRs (green), coding exons (blue), 3′ UTRs (red), and specific protein domains (purple, orange) in major transcript isoforms. Upper numbers in the sashimi plots indicate the inclusion counts (IJC) and lower numbers indicate the skipping counts (SJC) as described in Figure 3A. CK: control condition; LK: low K+ stress; IncLevel: inclusion level. [file Image_6.TIF]
